# Sexual dimorphism of sleep regulated by juvenile hormone signaling in *Drosophila*

**DOI:** 10.1371/journal.pgen.1007318

**Published:** 2018-04-04

**Authors:** Binbin Wu, Lingling Ma, Enyan Zhang, Juan Du, Suning Liu, Jeffrey Price, Sheng Li, Zhangwu Zhao

**Affiliations:** 1 Department of Entomology and MOA Key Lab of Pest Monitoring and Green Management, College of Plant Protection, China Agricultural University, Beijing, China; 2 Guangzhou Key Laboratory of Insect Development Regulation and Application Research, Institute of Insect Science and Technology & School of Life Sciences, South China Normal University, Guangzhou, China; 3 Department of Neurology and Cognitive Neuroscience, School of Biological Sciences, University of Missouri-Kansas City, Kansas City, Missouri, United States of America; Washington University in Saint Louis School of Medicine, UNITED STATES

## Abstract

Sexually dimorphic phenotypes are a universal phenomenon in animals. In the model animal fruit fly *Drosophila*, males and females exhibit long- and short-sleep phenotypes, respectively. However, the mechanism is still a mystery. In this study, we showed that juvenile hormone (JH) is involved in regulation of sexually dimorphic sleep in *Drosophila*, in which gain of JH function enlarges differences of the dimorphic sleep phenotype with higher sleep in males and lower sleep in females, while loss of JH function blurs these differences and results in feminization of male sleep and masculinization of female sleep. Further studies indicate that *germ cell-expressed* (GCE), one of the JH receptors, mediates the response in the JH pathway because the sexually dimorphic sleep phenotypes cannot be rescued by JH hormone in a *gce* deletion mutant. The JH-GCE regulated sleep dimorphism is generated through the sex differentiation-related genes -*fruitless* (*fru*) and *doublesex* (*dsx*) in males and *sex-lethal* (*sxl*), *transformer* (*tra*) and *doublesex* (*dsx*) in females. These are the “switch” genes that separately control the sleep pattern in males and females. Moreover, analysis of sleep deprivation and circadian behaviors showed that the sexually dimorphic sleep induced by JH signals is a change of sleep drive and independent of the circadian clock. Furthermore, we found that JH seems to also play an unanticipated role in antagonism of an aging-induced sleep decrease in male flies. Taken together, these results indicate that the JH signal pathway is critical for maintenance of sexually dimorphic sleep by regulating sex-relevant genes.

## Author summary

Sleep is a very important biological behavior in all animals and takes up around one third of the lifespan in many animals. In both insects and mammals (including humans), sleep differences between male and female (sexually dimorphic sleep) have been described over the past decades. However, its internal regulation mechanism is still unclear. The fruit fly *Drosophila melanogaster*, sharing most sleep characteristics with humans, has been used for sleep studies as a powerful model for genetic analysis. In this study, we reported that Juvenile hormone (JH) induces completely different sleep effects between males and females with higher sleep in males and lower sleep in females, while loss of JH function blurs these differences and results in feminization of male sleep and masculinization of female sleep. Further studies indicate that the sexual dimorphism of sleep is generated through the sex differentiation-related genes regulated by JH and its receptor GCE (germ cell-expressed) signaling. Furthermore, we found that JH seems to also play an unanticipated role in aging-induced sleep changes.

## Introduction

Sleep is a general phenomenon identified in many species and is usually accompanied with various sex-specific properties. For instance, in human beings, women have higher sleep spindle number and density than men[1]. Also there are sex differences in responses of male and female rats to sleep deprivation[2]. In recent years, the *Drosophila* genetic model system for sleep research has been shown to share all the key characteristics of mammalian sleep[3,4,5]. Similar to a variety of organisms, sex-specific properties in sleep pattern are present in fruit flies. Previous studies reported that there is a large sex difference in the total sleep amounts during the daylight hours, in which female sleep is only 40% of male sleep in *D*. *melanogaster*[6].To address that phenomenon, specific neurons[7,8] and non-neural factors [8] relevant to sexually dimorphic sleep have been discussed. Other types of sexual dimorphism (e.g., stress resistance, feeding, and physical characteristics) mediated by insulin, dopamine and Juvenile Hormone (JH) may participate in the relevant regulatory circuits[9,10], but their specific interactions and the molecular mechanism are still unclear. Here, we examine the role of JH in the control of a sexually dimorphic sleep phenotype.

Juvenile hormones are a group of acyclic sesquiterpenoids that regulate insect physiological processes, such as development, reproduction and diapause[11]. JHs are synthesized primarily in the *corpora allata* (CA), a pair of endocrine glands with neural connections to the brain[12]. The pars intercerebralis (PI) has been recently identified as a structure of neuroendocrine regulation for sleep and wakefulness in *Drosophila*[13]. It has been suggested that at least part of the CA is derived from the PIa/c (PIa: located around the medial lobes of the mushroom body; PIc: located dorsal to the central complex)[14]. Furthermore, JH could serve as a molecular link between PI neurons and the *corpus cardiacum—copora allata*(CC-CA) gland[15]. Previous studies mapped the PI area of the brain which feminizes male locomotor activity through up-regulated expression of the sex-determination gene *transformer* (*tra*)[16].

In *D*. *melanogaster*, the presence of XX (or XY) sex chromosome constitution promotes female (or male) somatic development through a sex determination hierarchy[17]. The binary switch gene *sex-lethal* (*sxl*) regulated by the X chromosome counting system controls sexual identity in *Drosophila*. When SXL is on, it imposes female development, whereas male development proceeds when it is off[18]. *Transformer* (*tra*), *fruitless* (*fru*) and *doublesex* (*dsx*) act downstream of *sxl* to control sexual development in *Drosophila*. TRA regulates female-specific alternative splicing of *dsx* to encode DSX^F^ protein. Without TRA, male-specific alternative splicing of *dsx* pre-mRNA occurs, and this transcript encodes DSX^M^ protein[19]. *Fru*, a pleiotropic gene which lies at the bottom of the sex-determination hierarchy, controls male sexual behavior and is essential for viability in *Drosophila*[20]. Indeed, these genes products control sexual differentiation of the organism body and also contribute to relevant behavioral and neural development[21].

Biosynthetic activity in the CA is considered to be a major factor in the regulation of JH titer[22,23]. The JH biosynthetic pathway mainly includes two steps: the generation of farnesyl diphosphate (FPP) via the mevalonate pathway in early steps, and then conversion of farnesoic acid (FA) generated from FPP to active JH in later steps[11], in which many enzymes have been found to be responsible for the biosynthesis of JH, such as HMG-CoA synthase (HMGS), HMG-CoA reductase (HMGCR), phosphomevalonate kinase (MevPK), and JH acid O-methyltransferase (JHAMT)[24,25]. Among these, the enzyme JHAMT expressed in the CA activates the JH biosynthesis pathway in the last step and is considered the key regulator in JH biosynthesis. In *D*. *melanogaster*, the JHAMT expression pattern is consistent with changes of the JH titer, and recombinant *Dm*JHAMT protein can produce JHs with the presence of its substrates[23]. In addition, the rate of JH in vitro biosynthesis by brain-ring gland complex is higher when JHAMT is over-expressed in the CA[26]. In *Bombyx mori*, *jhamt* mRNA level is consistent with JH titer, and the transcriptional suppression of *jhamt* gene is crucial for down-regulation of JH biosynthesis[11,25]. Therefore, JHAMT is a useful and important index for JH. In this study, we explored the role of JH and its signal pathway on sleep regulation through a genetically transgenic system.

## Results

### Effects of JH on sleep

**Fig 1:**
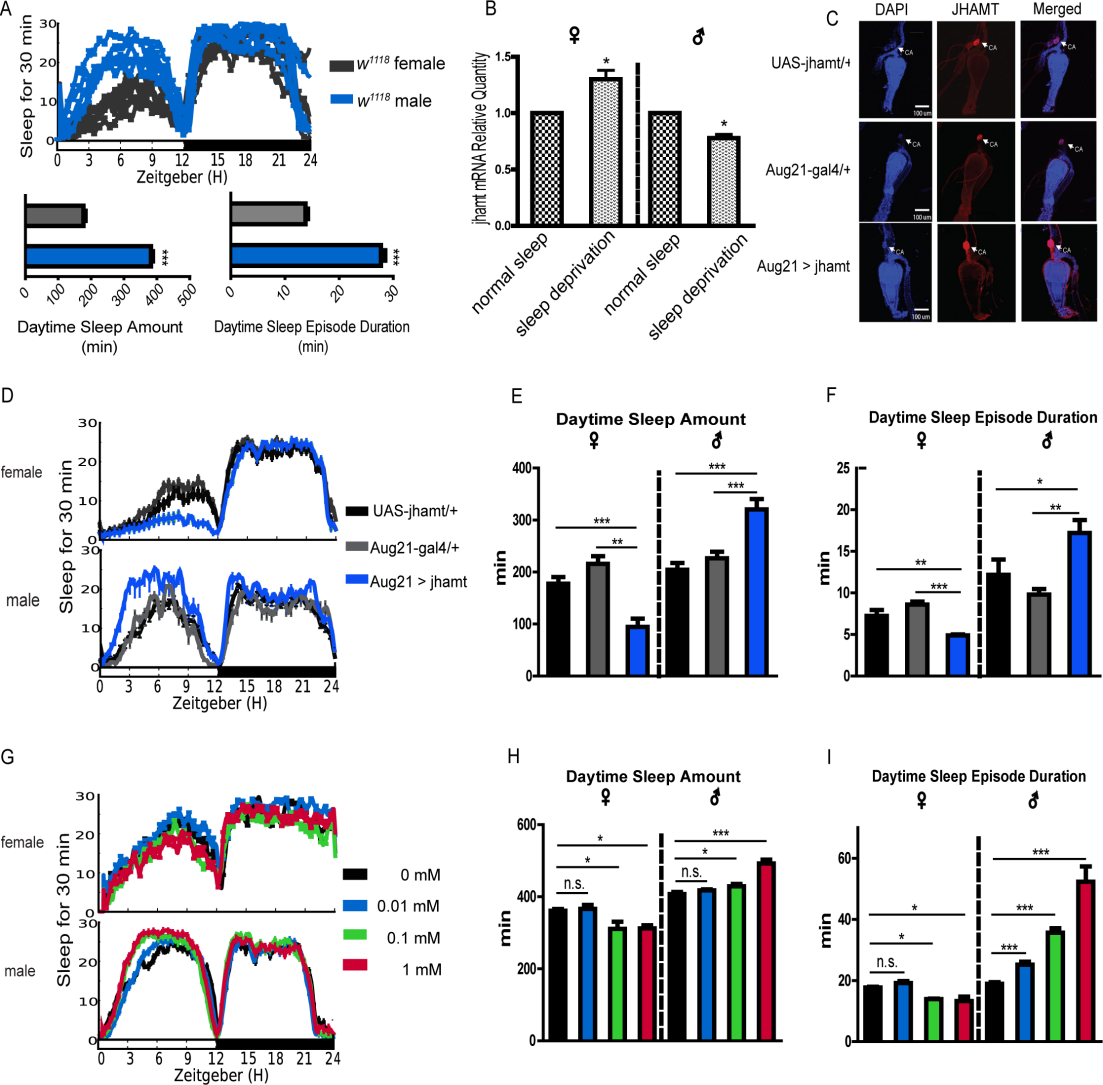
**Effects of gain of JH function on sleep behaviors**. (A) Sleep patterns of male and female flies; the gray represents *w*^*1118*^ female, and the blue represents *w*^*1118*^ male. Individual plots for multiple females and males are shown, in which one plot trace line represents a repeat with 16–32 flies. Tabulated data represent mean±SEM (n = 112–145).***P<0.001 determined by Student’s t test. (B) *Jhamt* relative expression in *w*^*1118*^ males and females after sleep deprivation. Data represent mean±SEM. *P<0.05 determined by Student’s t test. (C) Aug21-gal4 was used to drive JHAMT over-expression in the *corpora allata* cells. The blue represents DAPI, the red represents anti-JHAMT, and the third column represents co-location of DAPI and anti-JHAMT. (D) Sleep trace graph of flies over-expressing *jhamt* with Aug21-gal4. The dark and gray colors separately represent *UAS-jhamt* and *Aug21-gal4* as controls. The blue color represents *jhamt* over-expression.(E) Daytime sleep amount of flies over-expressing *jhamt* showed a significant decrease in the female but an increase in the male. Data represent mean±SEM (n = 32–96). **P<0.01, ***P<0.001 determined by Student’s t test. (F) Daytime sleep-bout duration of flies over-expressing *jhamt* showed a significant decrease in the female but an increase in the male. Data represent mean±SEM (n = 32–96). *P<0.05, **P<0.01, ***P<0.001 determined by Student’s t test. (G) Sleep trace graph of flies with the treatments of different concentrations of JH analog. The dark, blue, green and red lines represent 0mM, 0.01mM, 0.1mM and 1mM of JH analog, respectively. (H) Daytime sleep amount after administration of 0mM, 0.01mM, 0.1mM and 1mM JH analog to adult flies. Data represent mean±SEM (n = 32–96). *P<0.05, ***P<0.001 determined by Student’s t test. (I) Daytime sleep-bout duration after administration of 0mM, 0.01mM, 0.1mM and 1mM JH analog to adult flies. Data represent mean±SEM (n = 32–96). *P<0.05, ***P<0.001 determined by Student’s t test.

To compare sleep-wake profiles between both sexes, we monitored hundreds of wild type flies. Results showed that sleep of males during daytime was much higher than that of females (Fig 1A). Transcriptomic analysis of sleep-deprived and non-deprived rats has shown that sleep–wakefulness states affect gene expression[27]. Thus, we performed a sleep deprivation assay by forcing flies of wild-type *w*^*1118*^ to be awake all night using mechanical vibration, and surprisingly found that the changes of *jhamt* transcript levels exhibited two opposite tendencies in the male and female flies after 12 h sleep deprivation (Fig 1B). To further elucidate the function of endogenous JH signaling on the regulation of sleep in flies, we used a tissue-specific driver (Aug21-gal4) to over-express JHAMT—a key enzyme synthesizing JH in the CA cells[26,28]. Over-expression was confirmed by immunofluorescence with anti-*Dm*JHAMT antibody (Fig 1C). Results showed that flies with up-regulated JHAMT exhibited enhanced sleep differences between both sexes, in which the daytime sleep amount in males showed a significant increase but a significant decrease in females(Fig 1D and 1E), primarily caused by sleep episode duration (Fig 1F). Furthermore, in order to eliminate the possibility that abnormality of metamorphosis development would contribute to non-specific effects on sleep, we applied three different concentrations of exogenous pyriproxyfen–a juvenile hormone analog (JHA)- to adult wild-type flies, and then sleep behavior was monitored. Results showed that flies with JHA application exhibited similar sexually dimorphic sleep responses like those above with genetic gain of JHAMT function, with 0.1mM JHA as an effective concentration and regulation of sleep showing dose-dependency(Fig 1G–1I).

**Fig 2:**
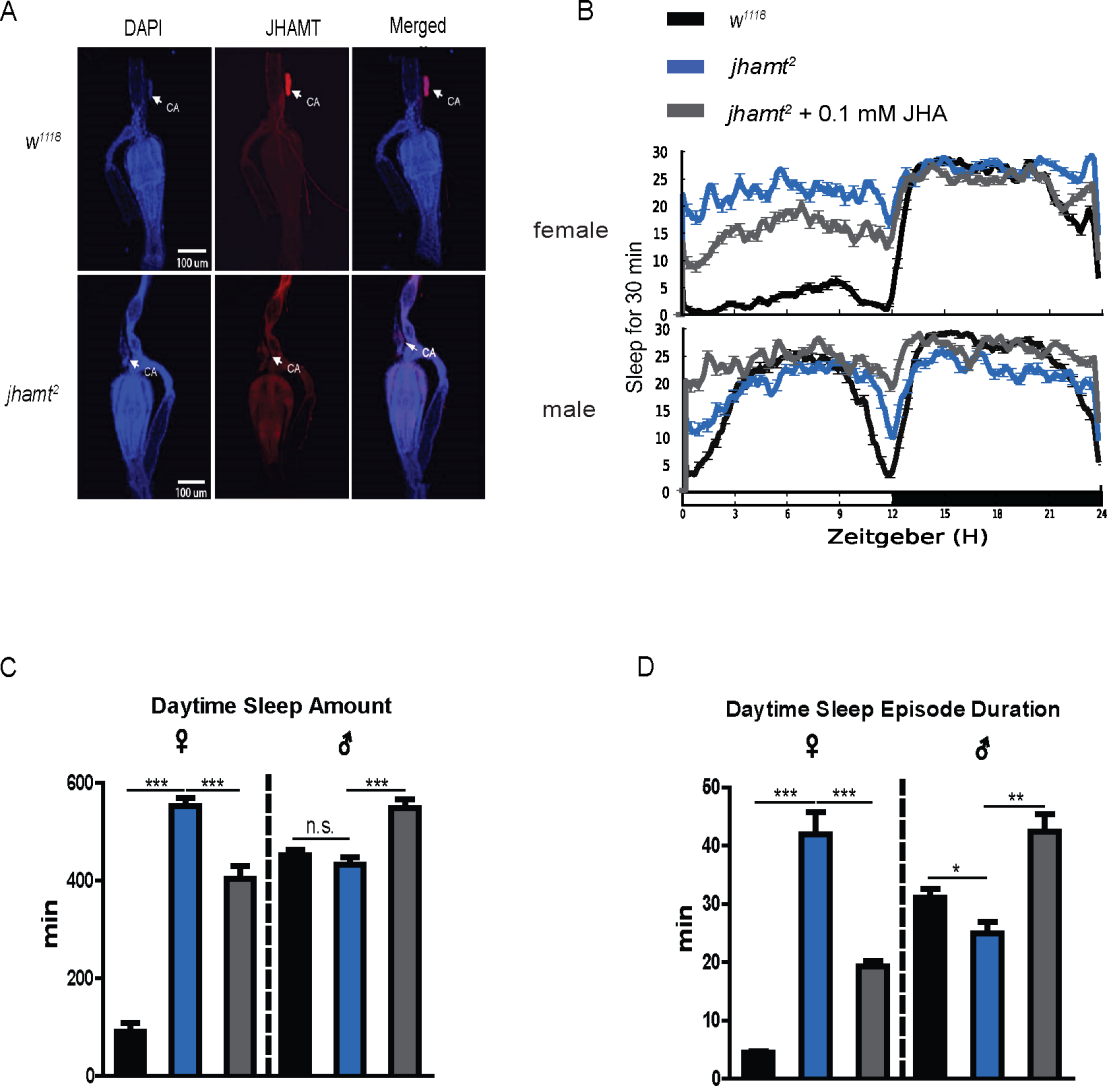
**Effects of *jhamt* mutation on sleep behaviors**. (A) JHAMT protein could not be detected in *jhamt*^*2*^ corpora allata cells. The blue represents DAPI, the red represents anti-JHAMT, and the third column represents a co-location of DAPI and anti-JHAMT. (B) Sleep trace graph of *jhamt*^*2*^ and JH analog-fed *jhamt*^*2*^ flies. The dark, blue and gray lines represent *w*^*1118*^, *jhamt*^*2*^ and 0.1mM JH analog-fed *jhamt*^*2*^ flies, respectively. (C) Daytime sleep amount of *jhamt*^*2*^ and JH analog-fed *jhamt*^*2*^ flies. With loss of *jhamt* function, daytime sleep amount in the female significantly increased, and the males had a slight but not significant decrease in daytime sleep amount. The effects of administration of JH analog were consistent with Fig 1D. Data represent mean±SEM (n = 32–96). n.s. represents no significant difference, ***P< 0.001 determined by Student’s t test. (D) Daytime sleep-bout duration of *jhamt*^*2*^ and JH analog-fed *jhamt*^*2*^ flies. The results were generally consistent with their daytime sleep, except that the sleep-bout duration of the male exhibited a significant difference between *w*^*1118*^ and *jhamt*^*2*^. Data represent mean±SEM (n = 32–96). *P<0.05, **P<0.01, ***P< 0.001 determined by Student’s t test.

For a loss-of-function analysis, we used a *jhamt* mutant (*jhamt*^*2*^, in which JHAMT is effectively deleted [29]), which was further confirmed by immunofluorescence with anti-*Dm*JHAMT antibody, to assess sleep regulation (Fig 2A). With loss of *jhamt* function, the daytime sleep amount in females significantly increased (Fig 2B and 2C). Although sleep amount of males during daytime showed no significant difference compared to control (Fig 2B and 2C), sleep quality was significantly affected by decreases of the sleep-bout duration (Fig 2D), indicating reduction of sleep quality in the mutant males. The daytime sleep amount and sleep-bout duration in the *jhamt*-deleted mutants of both males and females was completely or partially rescued by exposing them to 0.1mM JHA, in which sleep quality of males increased and of females decreased (Fig 2B–2D).

All these results above indicate that males and females exhibit a JH-responsive sexually dimorphic sleep phenotype—a long sleep phenotype in males and a short sleep phenotype in females. An increase of JH increases the sexually dimorphic sleep phenotype, and a decrease of JH decreases this difference and blurs the gender boundaries. In other words, lack of JH will result in feminization of male sleep and masculinization of female sleep.

### Effects of JH receptors on sleep

**Fig 3:**
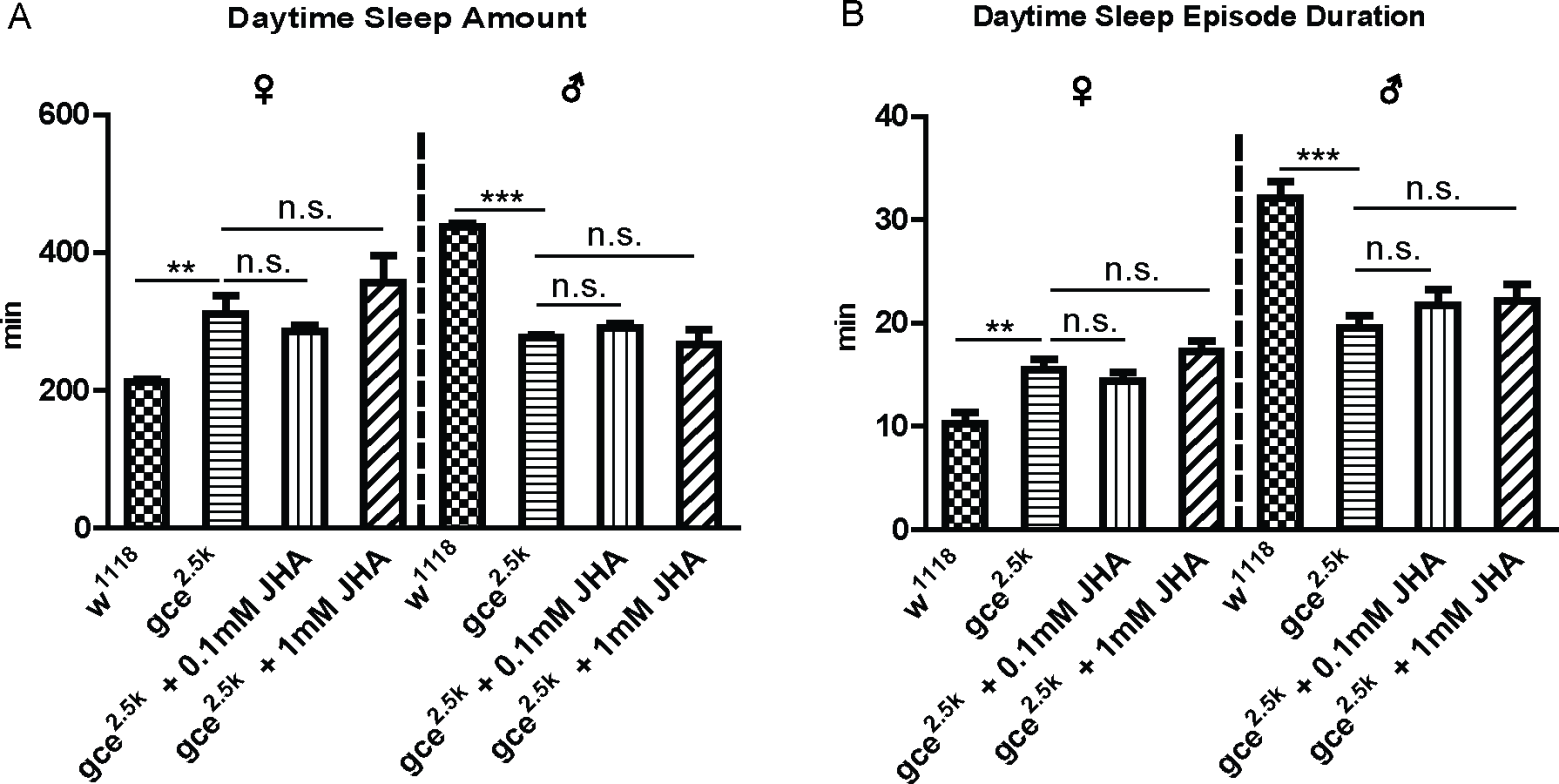
**Effects of JH receptor *gce* mutations on sleep behavior**. (A) *Gce* mutation increased the female daytime sleep amount and decreased the male daytime sleep amount, while neither 0.1mM nor 1mM JH analog could rescue the mutant phenotypes. Data represent mean±SEM (n = 32–96). n.s. represents no significant difference, **P<0.01, ***P< 0.001 determined by Student’s t test. (B) Sleep-bout duration of *gce*^*2*.*5k*^ flies was consistent with their daytime sleep amount. Data represent mean±SEM (n = 32–96). n.s. represents no significant difference, **P<0.01, ***P< 0.001 determined by Student’s t test.

The *Drosophila* germ cell-expressed (GCE) and methoprene-tolerant (MET), both belonging to bHLH-PAS transcription factors, are products of two paralogous genes on the X chromosome. Both proteins potentially mediate the effects of JH as candidate JH receptors[30]. In this study, *gce*^*2*.*5k*^ and *met*^*27*^ (both of them are null alleles) were used to further investigate the function of the JH signaling pathway on daytime sleep. Results showed that the *gce* deletion mutant also exhibited sexually dimorphic effects on sleep. *Gce* mutant male flies exhibited a short-sleep phenotype, while female flies exhibited a long-sleep phenotype. Moreover, administration of 0.1mM JHA did not rescue the sleep phenotypes, and even increasing JHA concentration to 1mM did not rescue the sleep phenotype (Fig 3A and 3B). However, in the *met* mutant, the males and females did not exhibit sexually dimorphic sleep phenotypes, but administration of 1mM JHA did effectively induce sexual dimorphism again (S1A and S1B Fig), indicating the *met* mutation may be partially involved in a JH-responsive sexually dimorphic sleep regulatory pathway, and it may be also involved in other signaling to affect flies’ daytime sleep. The sleep behavior of *Gce*^*2*.*5k*^ but not of *met*^*27*^ phenocopied *jhamt*^*2*^, suggesting that GCE is the main receptor to regulate *Drosophila* sexually dimorphic sleep in the JH signaling pathway.

### JH-GCE signaling regulates expressions of sex determination genes

**Fig 4:**
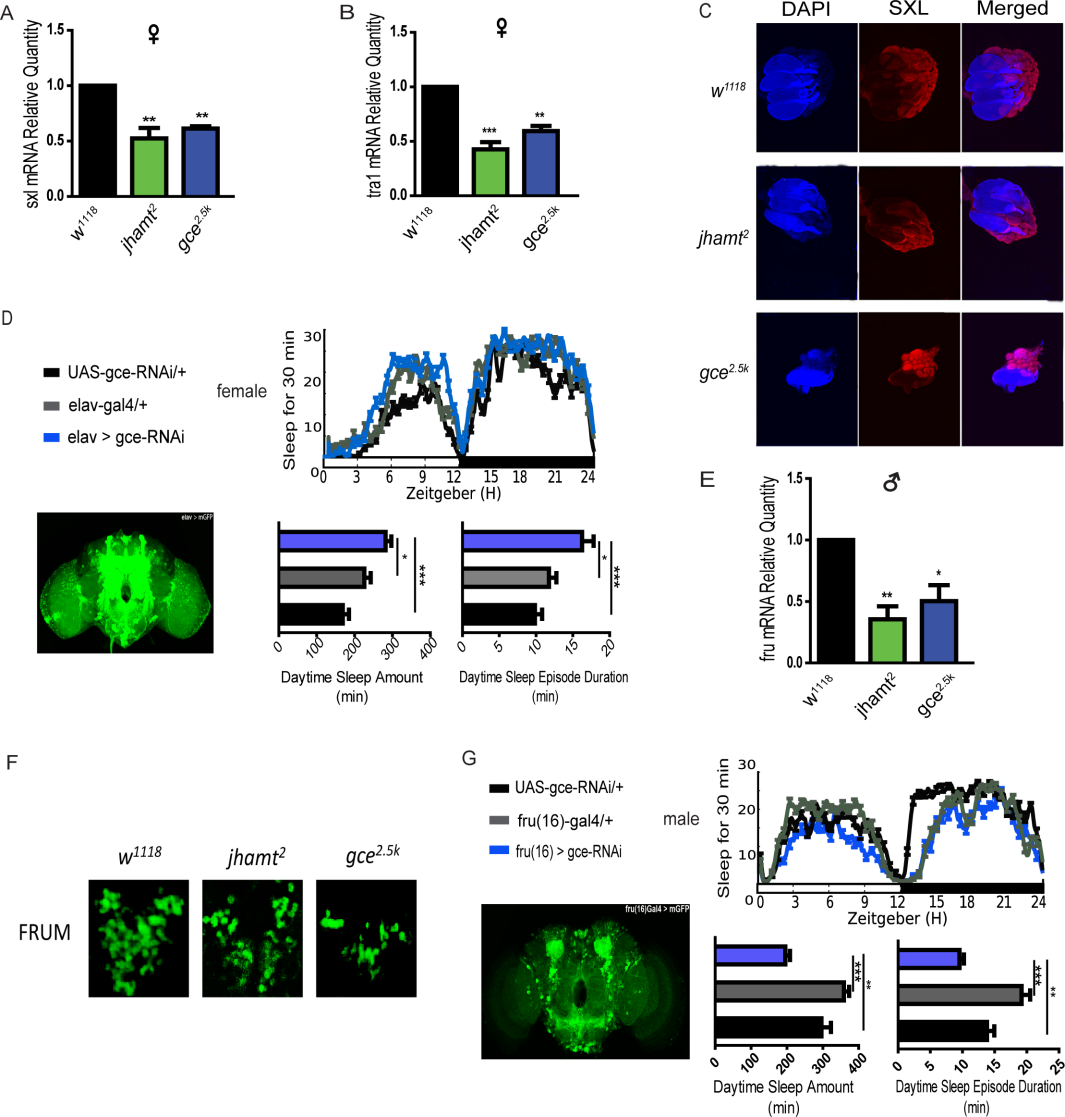
JH-GCE signaling pathway affected sex-related gene expression. (A, B and E) *Sxl*, *tra1* and *fru* relative transcription levels in *w*^*1118*^, *jhamt*^*2*^ and *gce*^*2*.*5k*^ female or male flies. Data represent mean±SEM. *P<0.05, **P<0.01, ***P< 0.001 determined by Student’s t test. (C) The protein expression of SXL in female ovary. First row: *w*^*1118*^; Second row: *jhamt*^*2*^; Third row: *gce*^*2*.*5k*^. The blue represents DAPI, the red represents anti-SXL, and the third column represents co-location of DAPI and anti-SXL. (D) Sleep analysis of female flies with *gce* dsRNA interference driven by *elav-gal4*. The color of dark, gray and blue represent *UAS-gce-RNAi*, *elav-gal4* (as controls) and *elav>gce-RNAi*, respectively. The line diagram is sleep trace graph; the two histograms are daytime sleep amount and daytime sleep-bout duration quantification. The bottom left figure shows the neurons with *gce* dsRNA interference, as detected with a GFP reporter. (F) FRU^M^ protein expression in pars intercerebralis of *w*^*1118*^, *jhamt*^*2*^ and *gce*^*2*.*5k*^. (G) Sleep status of male flies with *gce* dsRNA interference in fru(16)-gal4 neurons. The color of dark, gray and blue represent UAS-gce-RNAi, fru(16)-gal4 and fru(16)> gce-RNAi, respectively. The line diagram is sleep trace graph; the two histograms are quantified analysis of the sleep amount and sleep bout duration during daytime. The bottom left figure shows the neurons with *gce* dsRNA interference, as detected with a GFP reporter.

To determine how the JH pathway acts on sexual sleep differentiation, we focused on the JH-GCE signaling pathway effects on the sex determination. *Sex-lethal* (*sxl*), a key upstream gene of sex-determination in female flies, is well known to establish and maintain sex determination in females[31], while *Fru*^*M*^, a male-specific upstream gene for sex determination, has been shown to contribute to sexual dimorphism by sculpting the sexually dimorphic nervous system[32]. In this study, we compared mRNA levels from both female *sxl* and *tra* and male *fru*^*M*^ genes among *w*^*1118*^ (as control), *jhamt*^2^ and *gce*^*2*.5k^, and found their transcript levels in the *jhamt* and *gce* mutants almost decreased to half compared with control levels (Fig 4A, 4B & 4E). Because SXL is mainly expressed in female germline cells[33], we determined SXL protein expression in female ovaries of wild-type *w*^*1118*^, *jhamt*^2^ and *gce*^*2*.*5k*^. Results showed that SXL protein expression was much less in both *jhamt* and *gce* deletion mutants than in *w*^*1118*^ controls (Fig 4C). Abnormal expression of SXL in female flies suggested that the female sex differentiation pathway, mediated by SXL and TRA, may be affected in *jhamt* and *gce* mutants. We further used *elav-gal4* to drive *gce* dsRNA expression in a large field of central neurons and the results showed that the sleep pattern of female flies was much closer to that of males (Fig 4D). FRU^M^ protein is broadly expressed in the central nervous system of males, and the *gce* mutation led to reduced FRU^M^ protein in the PI neurons (Fig 4F), which have been reported to be involved in locomotor feminization[16]. Similarly, the density of FRU^M^ also decreased to a certain degree in *jhamt*^*2*^ males (Fig 4F). Furthermore, we specifically used a *fru* driver (*fru(16)-gal4*) to drive *gce* dsRNA expression and found the male flies’ daytime sleep significantly reduced as assessed by both the daytime sleep amount and the daytime sleep-bout duration (Fig 4G). These results indicate that JH-GCE pathway is important for regulating sexual determination genes and sleep behaviors via related neurons.

**Fig 5:**
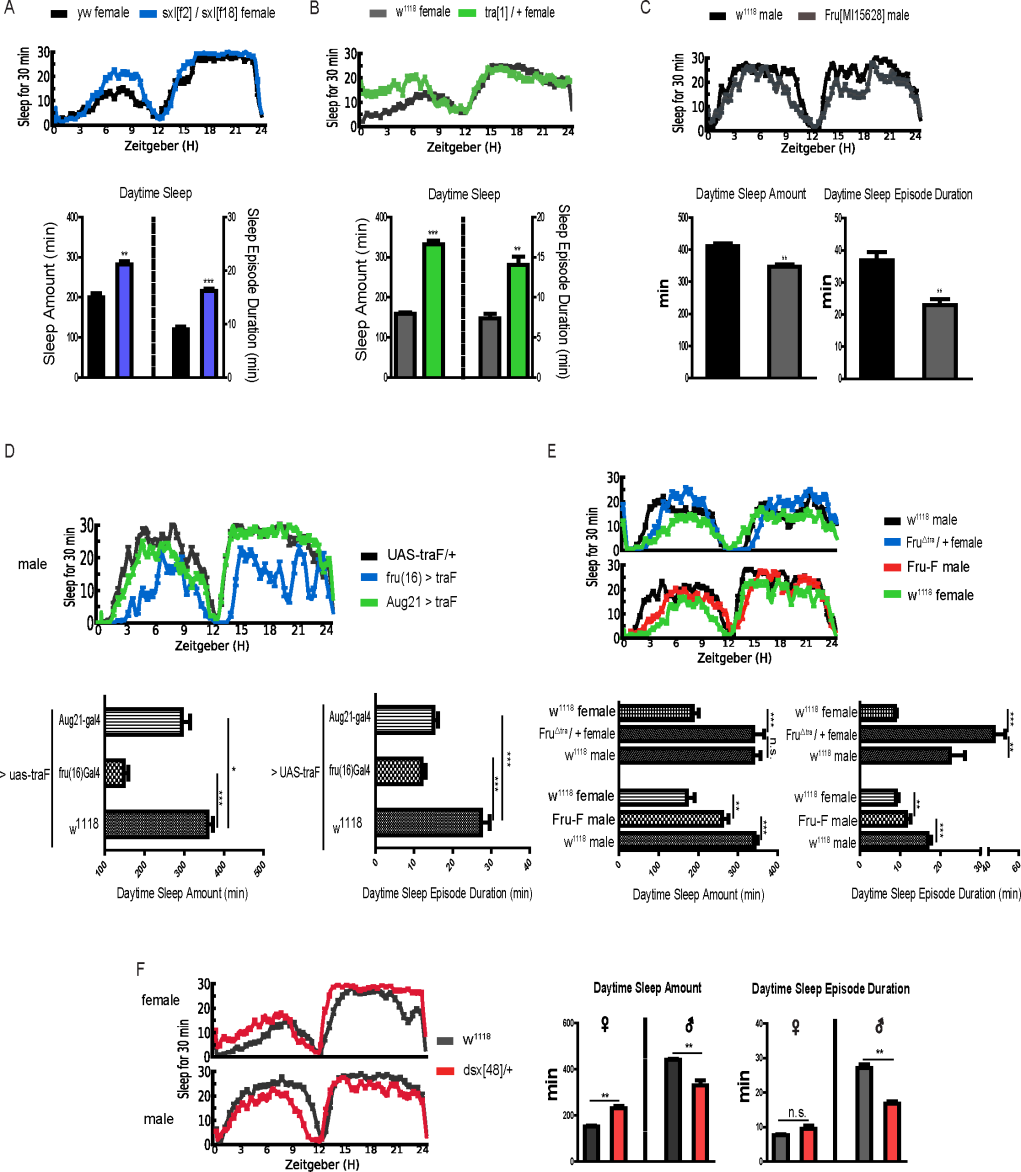
Flies with sex differentiation-related gene mutations phenocopied *jhamt*^*2*^ and *gce*^*2*.*5k*^ sleep behaviors. (A and B) The top graph is sleep trace; the bottom graph is quantified analysis of daytime sleep amount and sleep-bout duration. The color of black, blue, gray and green represent *yw*, *sxl[f2]*/*sxl[f18]*, *w*^*1118*^ and tra[1]/+ female flies, respectively. Data represent mean±SEM (n = 64–96). **P<0.01, ***P< 0.001 determined by Student’s t test. (C) Sleep trace, daytime sleep amounts and sleep-bout durations of *w*^*1118*^ and *fru[MI15628]* males. Data represent mean±SEM (n = 64–96). **P<0.01 determined by Student’s t test. (D) Sleep analysis of male flies with over-expression of traF driven by *fru(16)-gal4* and *Aug21-gal4*. The colors of black, blue and green lines represent *UAS-traF/+*, *fru(16) >traF* and *Aug21 >traF* sleep traces, respectively. The bottom graph is daytime sleep amount and sleep-bout duration. Data represent mean±SEM (n = 16–32). **P<0.01, ***P< 0.001 determined by Student’s t test. (E) The top picture shows sleep traces of *w*^*1118*^ males, Fru^Δtra^females, Fru-F males and *w*^*1118*^ females colored by black, blue, red and green, respectively. The bottom is quantified assays of daytime sleep amount and sleep-bout duration. Data represent mean±SEM (n = 32–64). **P<0.01, ***P< 0.001 determined by Student’s t test. n.s. represents no significance. (F) Sleep status of *w*^*1118*^ and dsx[48]/+ colored by gray and red, respectively. Data represent mean±SEM (n = 32–64).**P<0.01determined by Student’s t test. n.s. represents no significance.

To further identity the function of *sxl*, *tra* and *fru* genes in sleep regulation, we separately used deletion or insertion mutants blocking expression of *sxl*, *tra* and *fru* to observe effects on sleep behavior. Trans-heterozygous mutant sxl[f2]/sxl[f18] females are viable and fertile, but their sleep pattern is similar to the male pattern with an increased sleep during daytime (Fig 5A), and heterozygous *tra* mutants (tra[1]/+) also exhibit increased sleep in females (Fig 5B). In contrast, a *fru* insertion mutation in males caused significant decreases of sleep during the daytime (Fig 5C).

To verify the effects of masculinization or feminization, we simultaneously used different drivers (*Aug21-gal4 and Fru(16)-gal4*) to drive the expression of *traF* (Female-specific protein TRA) in males. Results revealed that male fly’s daytime sleep pattern is closest to the female fly’s daytime sleep when the *traF* of females is expressed in *fru* neurons of males, which is consistent with the importance of FRU-expressing neurons for the male sleep pattern. Expression of traF in the JH synthesis tissue *corpus allata* (with Aug21-Gal4) also significantly decreased daytime sleep amount and sleep bout duration of the male flies (Fig 5D). As for more genetic methods, Fru^Δtra^, with a modified S exon with *tra* binding sites mutation, produces female flies exhibiting a male sleep pattern (Fig 5E), and Fru-F, with a deletion of the S exon of the *fru* male specific splice form, produces male flies exhibiting a female sleep pattern (Fig 5E). Taken together, these results indicate that JH-GCE signaling regulating sleep sexual dimorphism is *via* the sexual determination genes *sxl-tra* in females and *fru* in males, respectively, and these genes seem to be the “switch” genes in females and males to separately control the sexually dimorphic sleep pattern in *D*. *melanogaster*.

Then, we explored another sex differentiation downstream gene by using a *dsx* heterozygous mutant. Previous research demonstrated this gene is bifunctional, since male-specific DSX represses female sexual differentiation and vice versa for female-specific DSX[34]. Our results showed that mutation of *dsx* in both males and females results in an intersexual sleep pattern. Total daytime sleep decreased about 22.5% (P < 0.01) in *dsx*[48]/+males and increased about 34.0% in females (P < 0.01)(Fig 5F), respectively. These results indicate that *dsx* is important to maintain sleep sexual differentiation. Thus, all sleep analysis in the context of sex differentiation-related genes suggests that JH signaling regulates sleep sexual dimorphism through sex differentiation-related genes.

### The JH-GCE signaling pathway is not related to sleep homeostasis and the circadian clock

**Fig 6:**
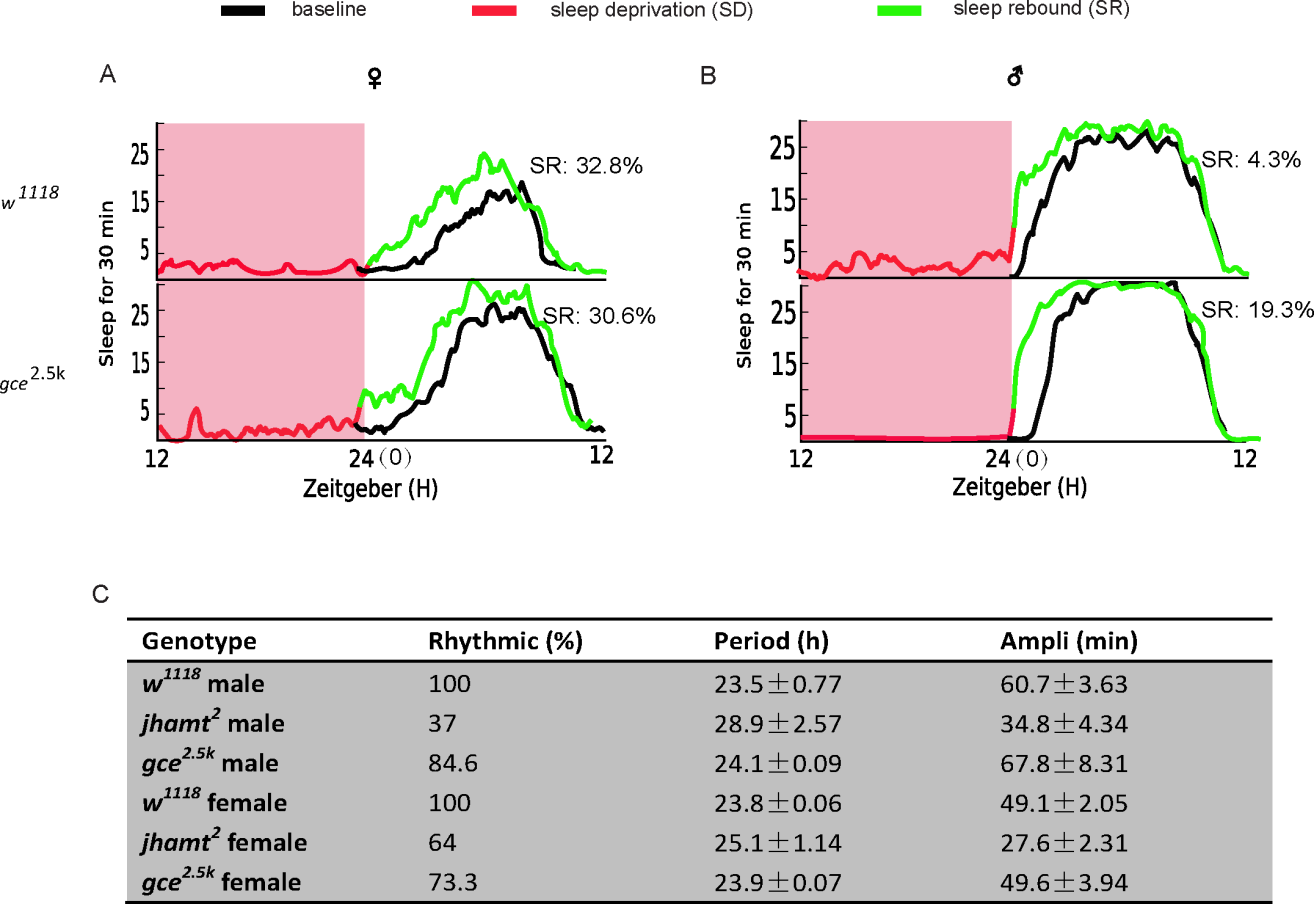
**Sleep homeostatic system was completely preserved in *gce*^*2*.*5k*^ flies**. (A and B) The *w*^*1118*^ and *gce*^*2*.*5k*^ flies exhibit sleep rebound after sleep deprivation. The black line represents sleep baseline. The red line (ZT 12 -ZT 24) represents sleep deprivation. The green line (ZT0-ZT12) represents sleep rebound. (C) Circadian phenotypes of *w*^*1118*^, *jhamt*^*2*^and *gce*^*2*.*5k*^. Data represent mean±SEM (n = 32–96).

Sleep is usually under control of homeostasis, which responds to sleep need and adjusts sleep intensity, and of the sleep time keeper, or the circadian clock[35,36]. Prolonged wakefulness increases sleep pressure which results in a rebound in the next sleep stage[37], and vice versa. To examine whether the homeostatic system is destroyed by a blocked JH signaling pathway, sleep deprivation was performed during the nighttime and sleep parameters of the next daytime were analyzed using the JH receptor mutant *gce*^*2*.*5k*^ and wild-type *w*^*1118*^ control flies. Results showed that a functional sleep homeostatic system was completely preserved in the *gce* mutant (Fig 6A and 6B). We further examined the circadian parameters of *jhamt* and *gce* mutants (Fig 6C) and found that the *jhamt* mutation dampened the flies’ rhythm amplitude and rhythmicity. Furthermore, the *gce*^*2*.*5k*^ mutant still exhibited a robust activity rhythm and ~24h periodicity. Based on the circadian phenotypes of *jhamt*^*2*^ and *gce*^*2*.*5k*^, we reasoned that the JH-GCE signaling effects on sleep are circadian rhythm independent.

## Discussion

### Contributions of juvenile hormone, insulin, ecdysone and sex peptide signaling to sexually dimorphic sleep

**Fig 7:**
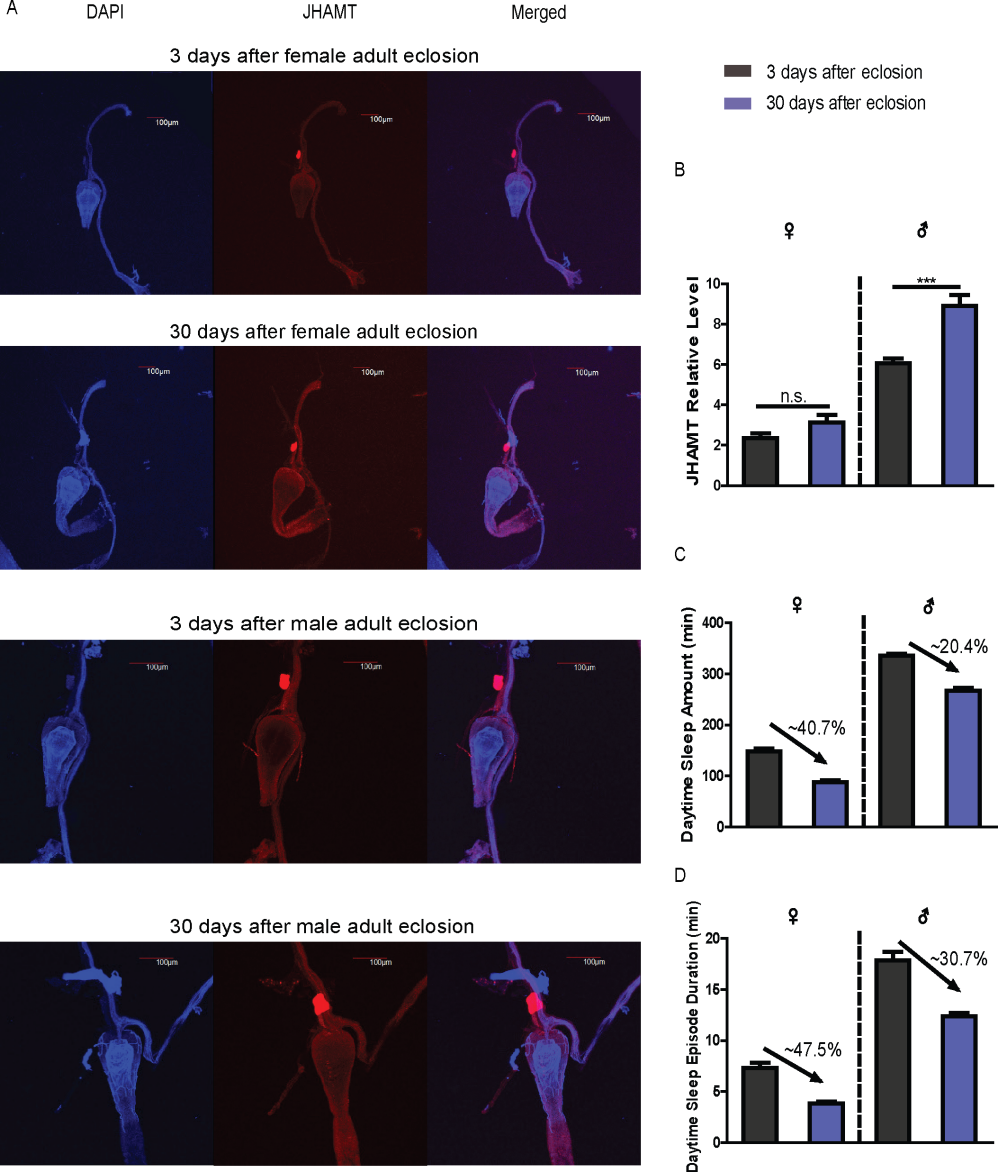
**Contributions of JH to sleep decrease in flies’ aging process**. (A) JHAMT expression in 3^rd^ day adults and 30^th^ day adults. The first row: 3^rd^ day female; second row: 30^th^ day female; third row: 3^rd^ day male; fourth row: 30^th^ day male. The blue represents DAPI, the red represents anti-JHAMT, and the third column represents co-localization of DAPI and anti-JHAMT. (B) Quantification of Fig 7A. The gray column represents 3^rd^ day flies, the blue column represents 30^th^ day flies. Data represent mean±SEM (n = 5–10). (C and D) Sleep changes in both sexes in 3^rd^ day adults and 30^th^ day adults which are colored with gray and blue. Data represent mean±SEM (n = 32–64).

The insulin system has been reported to be involved in regulation of sleep and locomotor activity [38,39]. A previous study showed that HMGCR, an enzyme responsible for the biosynthesis of JH, is controlled by the insulin receptor (lnR)[40]. A JH inhibitor fluvastain fed to male flies led to male locomotor activity feminization, and this effect was reversed by application of a JH analog[15]. Ablation of insulin-producing neurons in the adult PI or an insulin receptor mutant were also found to abolish sexual dimorphism in locomotor activity, suggesting that the insulin signaling pathway may work in conjunction with the JH pathway to control sexually dimorphic behaviors[38]. Ecdysone, another major endocrine hormone, has been shown to regulate adult flies’ daytime sleep[41]. However, the effects of ecdysone on sleep did not involve sex differences, although there is a vital relationship between JH and ecdysone for controlling molting and metamorphosis. As a male specific product, sex peptide inhibits female flies’ siesta sleep through copulation behavior, contributing to sexually dimorphic sleep[6]. Recently another paper reported that the male’s dorsal clock neurons are more active than those in females, in correlation with the increased sleep behavior of male flies[7]. In this study, we found that wild-type males synthesized more JHAMT protein in CA cells than females, and notably old flies (30^th^ day adults) seem to synthesize more JHAMT than young adults (3^rd^ day adults), although it is a small increase in females (Fig 7A and 7B). According to our sleep behavior results, increased JHAMT should promote male sleep and inhibit female sleep. Thus, the results imply that JH may act against sleep decrease in the male aging process. Interestingly, we did find that old male flies exhibit less sleep loss than old females, whose sleep loss reaches almost 50% (Fig 7C and 7D). These phenotypes possibly indicate that aging-induced sleep loss may include effects of JH.

### Role of JH receptors in sleep regulation

JH is an important endocrine hormone, which regulates many physiological and developmental processes through JH receptors. As the JH receptors, MET or/and GCE independently act in different functions[42]. For example, JH regulates the production of female sex pheromones via MET rather than its paralog GCE[43]. However, JH regulates nuclear receptor gene E75A in S2 cells through GCE but not MET[44]. According to the sleep behavioral assays of *gce* and *met* mutants, there is no doubt that GCE is the principal receptor for regulation of sexual dimorphism in sleep. Alternatively, *met* mutation caused sleep length to be significantly longer during the daytime in both sexes, suggesting that *met* may be involved in other regulatory pathways. A previous study reported that a MET/CYC heterodimer activates circadian rhythm-dependent gene expression in female mosquitoes[45], suggesting that *met* may control the circadian rhythm independent of sleep.

### Sex determination genes work in collaboration with the JH signal pathway in sleep regulation

From humans to insects, morphological and behavioral differences between male and female are ubiquitous. Mating, courtship-related sensory responses and aggression have been demonstrated to be under the control of the sex determination hierarchy[46,47,48]. *Tra* initiated by *sxl* modulates or even drives various female characteristics. A recent paper has reported that *myc* and *tra* can promote sexual size dimorphism in fruit flies[49]. In fact, the idea that the sex determination system participates in the regulation of sex-specific size in stag beetles was proposed several years ago, and JH was also shown to play a crucial role[10]. With regard to behavior, TRA can feminize male locomotor behavior in transgenic flies, as described above (Ref. 15). *Fru*, a terminal gene of the sex determination hierarchy, orchestrates male-specific neuronal morphology and behavior[50]. Activation of male-specific *dsx/fru* positive P1 neurons in female brain induces male-like courtship behavior[51]. Another terminal gene (*dsx*) coupled with *fru* to regulate male sexual behavior[52]. All these sex determination genes orchestrate the characteristics of “maleness” and “femaleness”. In this study, we showed that sexually dimorphic sleep in *Drosophila* regulated by JH-GCE signaling is produced by the pathway of sex-specific regulatory genes differing between males and females.

### A model of sexually dimorphic sleep regulated by the JH signal pathway in Drosophila

**Fig 8:**
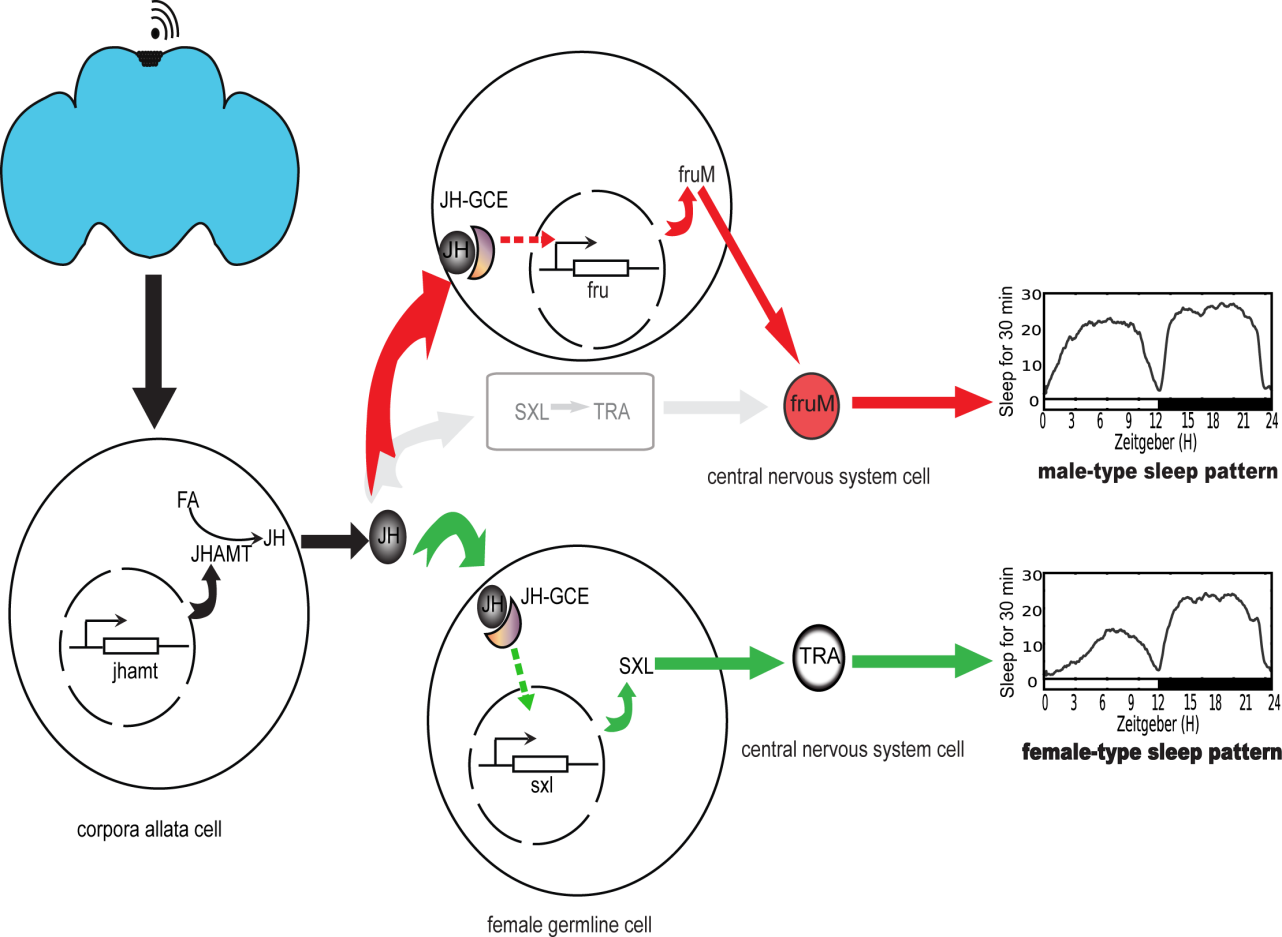
Model of JH-GCE signals regulating sexually dimorphic sleepJH signals from Drosophila brain (PI possibly) target sex differentiation-related genes and sustain male- and female-sleep types. The black arrow represents JH signals, while the red and green arrows represent JH/GCE-FRU and JH/GCE-SXL-TRA pathways, respectively.

We propose a model based on the available evidence to explain this complicated sleep sexual dimorphism phenomenon (Fig 8).The pars intercerebralis sends related signals to the *corpora allata*. The latter initiates *jhamt* transcription and activates JH biosynthesis from FA. JH is released and binds to the GCE protein to promote female-specific gene transcription in female *D*. *melanogaster*. On the other hand, JH signaling acts on *fru*, which encodes male specific protein FRU^M^ in the CNS, to maintain the male sleep pattern in the absence of SXL and TRA. DSX may be also responsible for sustaining male and female sleep behaviors. Therefore, the JH signaling pathway plays a necessary role in regulation of sexual dimorphism of sleep in *D*. *melanogaster*.

## Materials and methods

### Fly strains

The following strains were used in this study: UAS-*jhamt*, UAS-*gce*-RNAi, Aug21-gal4, *jhamt*-gal4, *jhamt*^2^, *gce*^2.5k^ and *met*^27^, which were previously reported (Ref. 26, Ref. 29 and Ref. 30). UAS-traF, fru(16)-gal4, Fru^Δtra^ and Fru-F were obtained from Yi Rao, Yufeng Pan and Chuan Zhou’s labs, respectively. *Dsx*[48]/TM2, *tra*[1]/TM2, *Sxl*[f2]/FM7c and *Sxl*[f18]/FM7c were purchased from the Bloomington Drosophila Stock Center. Flies were reared at 25°C and 65% relative humidity on a standard cornmeal-yeast-agar medium in a 12h light/12h dark cycle.

### Sleep behavioral assays

Three- to five-day-old flies were housed in monitor tubes (5[W] × 65[L] mm) with fly food. Experiments were performed in an incubator at a temperature of 25 ± 1°C and a relative humidity of 65%. Light was turned on at ZT0 (local time 06:30) and off at ZT12 (local time 18:30). The sleep activity was recorded using the *Drosophila* Activity Monitoring System (Trikinetics, Waltham, MA). The details for the experimental protocol and data analysis were described by Chen et al (2013).

Sleep was deprived by mechanical vibration of three seconds within each minute interval for the whole night (12h). All the sleep deprived flies were immediately fixed in liquid nitrogen for qRT-PCR assays.

### JHA treatment

The juvenile hormone analog (JHA) pyriproxyfen (gift from Xiru Liang, China Agriculture University) was dissolved in 95% ethanol, and JHA-containing food was prepared by adding JHA solution to standard *Drosophila* food at 50–55°C to the indicated concentrations. Newly eclosed flies were placed in glass tubes with standard medium for three days and transferred to detecting tubes with food containing JHA for behavior monitoring.

### Immunofluorescence

Three-day-old flies were fixed in 4% paraformaldehyde for 2 h. Then 6 to 8 flies were dissected in phosphate-buffered saline (PBS). They were washed 3 times with PBST (0.5% Triton X-100 in PBS) at room temperature. The tissue was soaked in blocking solution PNT (1% goat serum in PBS) for 1 h at room temperature and incubated with primary antibody for 16–24 h at 4°C. After 3 washes, the tissue was incubated in secondary antibody for 2 h at room temperature. The samples were analyzed with Nikon Eclipse TE2000-E and Nikon D-Eclipse (Nikon, Japan) confocal microscopes. Anti-DmJHAMT antibody was provided by Dr. Niwa. Anti-FruMale antibody was provided by the Yamamoto lab. Anti-SXL antibody was provided by DSHB at the University of Iowa.

### RNA Isolation, cDNA synthesis and Quantitative real-time PCR

RNA was isolated from whole bodies of 3-day-old male and female flies using Trizol Reagent (TIANGEN, Beijing) according to the manufacturer’s protocol. RNA was reverse transcribed using Fast cDNA Reverse Transcription Kit (TIANGEN, Beijing). The Quantitative real-time PCR assay was performed using Applied Biosystem Step One Real Time PCR system (Applied Biosystem, Foster, CA, USA), RealMasterMix kit and SuperReal PreMix Plus kit (TIANGEN, Beijing). The sequences of primers are shown in Supplementary S1 Table.

### Statistics analysis

Statistical analysis was performed with SPSS statistics 17.0. P values was obtained with *t*-test and considered significant at P < 0.05 and extremely significant at P < 0.001.

## Supporting information

S1 Fig Sleep behaviors of *met^27^* flies. (A) *Met* mutation led to increases in fly daytime sleep amount in both females and males. The third column represents *met*^*27*^ flies fed with 1mM JH analog. Data represent mean±SEM (n = 96). n.s. represents no significant difference. **P<0.01, ***P<0.001 determined by Student’s t test.
(B) Sleep-bout duration of *met*^*27*^ flies was consistent with their daytime sleep amount. And administration of 1mM JH analog induced a sleep sexual dimorphism. Data represent mean±SEM (n = 96). n.s. represents no significant difference. *P<0.05, **P<0.01, ***P<0.001 determined by Student’s t test.
(C) Circadian phenotypes of *met*^*27*^. Data represent mean±SEM (n = 32).
(TIF)

S1 Table qRT-PCR primers used in this study. (DOCX)
