# Supplementary figures and images for: Sexual dimorphism of sleep regulated by juvenile hormone signaling in Drosophila
Source: PLoS Genet. 2018 Apr 4;14(4):e1007318. doi: 10.1371/journal.pgen.1007318 (PMC5909909; doi:10.1371/journal.pgen.1007318)

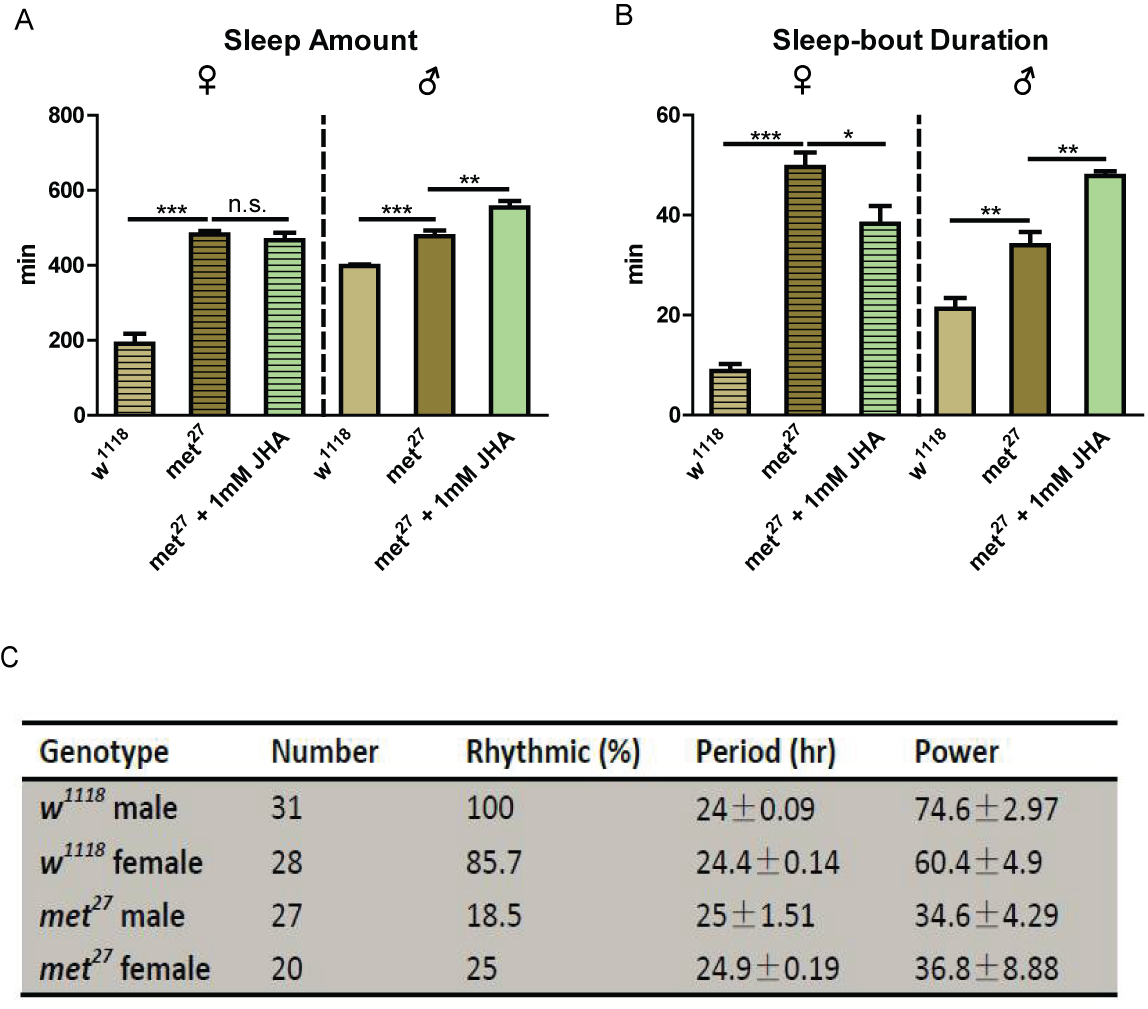

Supplement: S1 Fig — (A) Met mutation led to increases in fly daytime sleep amount in both females and males. The third column represents met27 flies fed with 1mM JH analog. Data represent mean±SEM (n = 96). n.s. represents no significant difference. **P<0.01, ***P<0.001 determined by Student’s t test. (B) Sleep-bout duration of met27 flies was consistent with their daytime sleep amount. And administration of 1mM JH analog induced a sleep sexual dimorphism. Data represent mean±SEM (n = 96). n.s. represents no significant difference. *P<0.05, **P<0.01, ***P<0.001 determined by Student’s t test. (C) Circadian phenotypes of met27. Data represent mean±SEM (n = 32). (TIF) [file pgen.1007318.s001.tif]
