# Supplementary material for: Sexual dimorphism of sleep regulated by juvenile hormone signaling in Drosophila
Source: PLoS Genet. 2018 Apr 4;14(4):e1007318. doi: 10.1371/journal.pgen.1007318 (PMC5909909; doi:10.1371/journal.pgen.1007318)
Supplement: S1 Table — (DOCX) [file pgen.1007318.s002.docx]

**Table S1: qRT-PCR primers used in this study**

| sxl-F | ACAAGCGTGAGGAGGC |
| --- | --- |
| sxl-R | CCCATCTGCGACATAAAG |
| tra1-F | GGAACCCAGCATCGAGATTC |
| tra1-R | ATCGCCCATGGTATTCTCTTTC |
| fru-F | GGGCGAAACAGTCAAGGC |
| fru-R | CAAGTAGATGATGGGATGTGGAT |
